# Translational impacts of enzymes that modify ribosomal RNA around the peptidyl transferase centre

**DOI:** 10.1080/15476286.2024.2368305

**Published:** 2024-07-01

**Authors:** Letian Bao, Josefine Liljeruhm, Rubén Crespo Blanco, Gerrit Brandis, Jaanus Remme, Anthony C. Forster

**Affiliations:** a Department of Cell and Molecular Biology, Uppsala University, Uppsala, Sweden; b Department of Molecular Biology, University of Tartu, Tartu, Estonia

**Keywords:** Modification enzymes, ribosomal RNA, peptidyl transferase centre, *E. coli*,translation

## Abstract

Large ribosomal RNAs (rRNAs) are modified heavily post-transcriptionally in functionally important regions but, paradoxically, individual knockouts (KOs) of the modification enzymes have minimal impact on *Escherichia coli* growth. Furthermore, we recently constructed a strain with combined KOs of five modification enzymes (RluC, RlmKL, RlmN, RlmM and RluE) of the ‘critical region’ of the peptidyl transferase centre (PTC) in 23S rRNA that exhibited only a minor growth defect at 37°C (although major at 20°C). However, our combined KO of modification enzymes RluC and RlmE (not RluE) resulted in conditional lethality (at 20°C). Although the growth rates for both multiple-KO strains were characterized, the molecular explanations for such deficits remain unclear. Here, we pinpoint biochemical defects in these strains. *In vitro* fast kinetics at 20°C and 37°C with ribosomes purified from both strains revealed, counterintuitively, the slowing of translocation, not peptide bond formation or peptidyl release. Elongation rates of protein synthesis *in vivo*, as judged by the kinetics of β-galactosidase induction, were also slowed. For the five-KO strain, the biggest deficit at 37°C was in 70S ribosome assembly, as judged by a dominant 50S peak in ribosome sucrose gradient profiles at 5 mM Mg^2+^. Reconstitution of this 50S subunit from purified five-KO rRNA and ribosomal proteins supported a direct role in ribosome biogenesis of the PTC region modifications *per se*, rather than of the modification enzymes. These results clarify the importance and roles of the enigmatic rRNA modifications.

## Graphical Abstract

**Figure:**
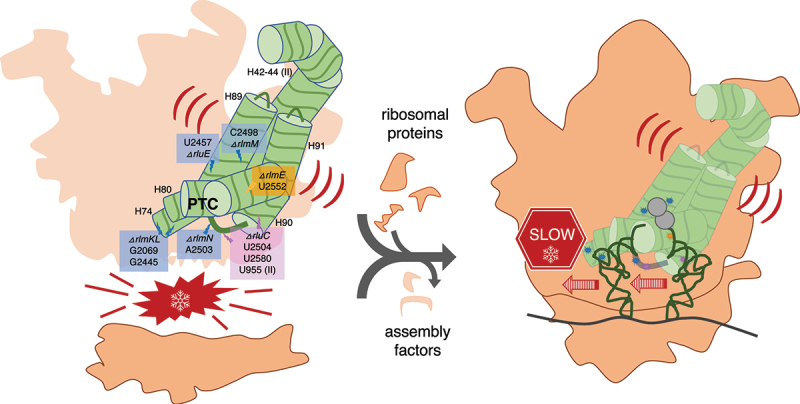

## Introduction

**Figure 1.**
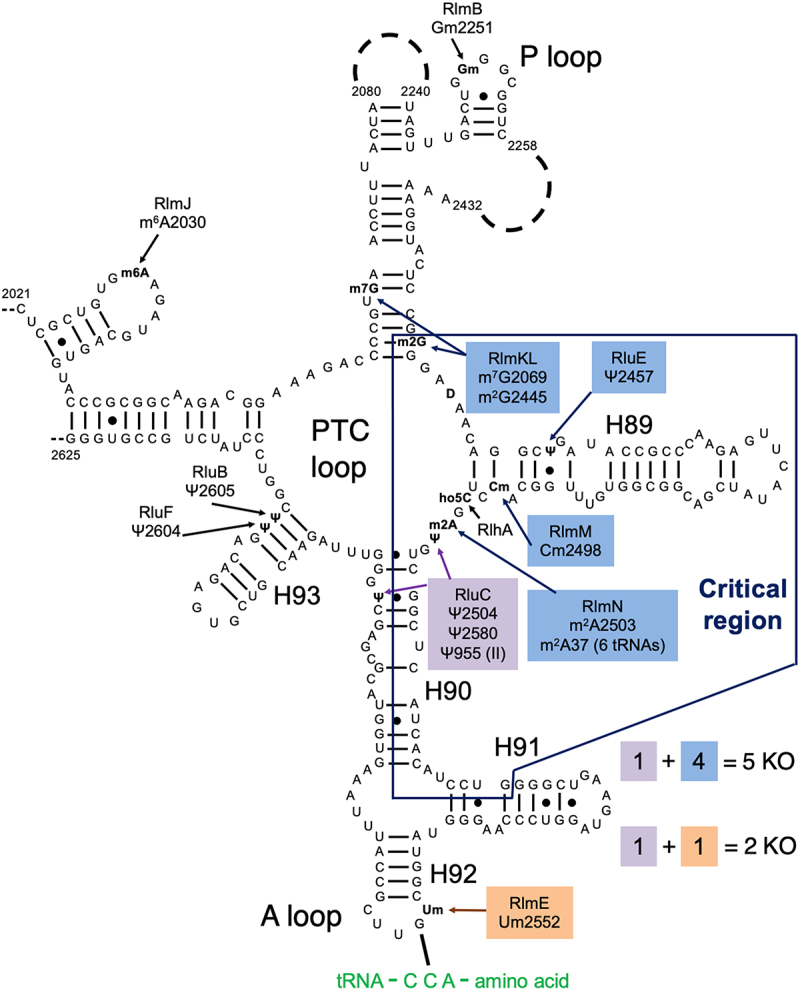
Our combination KOs of key rRNA modification enzymes for E. coli. The secondary structure contains most of domain V of the 23S rRNA, including the ‘critical region’ (hollow box) around the PTC loop. Shown are all domain V modifications (ho5C is partial) and the modification enzyme KOs used in this study (filled boxes). Base pairing with aminoacyl-tRNA at the A site is shown. Modified from Liljeruhm et al. (2022) with permission.

The ribosome is a complex ribonucleoprotein factory for cellular protein synthesis, termed 70S in *E. coli*. Initiation is carried out by the ribosomal small subunit, the 30S. It contains 16S rRNA that is modified posttranscriptionally, but the modifications have little effect on *in vitro* reconstitution and activity [1]. Both elongation and termination involve nucleophilic attacks catalysed by the ribosomal large (50S) subunit. It contains unmodified 5S ribosomal RNA (rRNA) and 23S rRNA that is heavily modified, mostly by pseudouridylations and methylations [2,3]. Thirteen of these 25 modifications are clustered in domain V around the peptidyl transferase centre (PTC) loop (Figure 1), suggesting functional importance (reviewed in [4,5]). However, the impacts of rRNA modifications on ribosome biogenesis, assembly and function remain poorly understood.

Individual knockouts (KOs) of all rRNA modification enzymes resulted in very minimal growth deficits (e.g. [6,7]). The notable exception, displaying a two- to fourfold decrease in growth rate, was *ΔrlmE* which abolished the 2’O-methylation of U2552 at the A site’s A loop adjacent to the PTC loop (Figure 1) [8]. An additional complication with KO studies is that non-modification ‘moonlighting’ functions of the enzymes are possible. Indeed, *ΔrlmE* was partially rescued by overexpressing the assembly factors EngA or ObgE [9]. But a catalytically inert RlmE mutant did not rescue the *ΔrlmE* growth deficit [10], implying a direct role for modification in growth. Moreover, depletion of SAM impacts ribosome biogenesis through hypomodification of Um2552 [11]. Biochemically, *ΔrlmE* ribosomes exhibited deficits in 70S assembly [9,12,13] and *in vitro* translocation [14]. The *ΔrlmE* strain accumulated ribosomal 45S intermediates with reduced levels of ribosomal protein [8], including L5 and L19 that form intersubunit bridges between the 50S and 30S subunits [15].

Due to the redundancy of individual bacterial rRNA modification enzymes, combined KOs are necessary to unmask their significance. In an *in vitro* study, Green and Noller [16] reported a ‘critical natural element’ of the 23S rRNA, an 80-nucleotide region around the PTC loop where modifications were essential for function *in vitro* (>100× stimulation; Figure 1). This was discovered by reconstituting 50S subunits with hybridized pairs of *in-vitro*-transcribed and isolated natural 23S rRNA portions and assaying for peptidyl transferase activity. This unphysiological assay, termed ‘the fragment reaction’, used a 5’-truncated fMet-tRNA^fMet^ fragment as the P-site substrate for the 50S, puromycin as the A-site substrate, and incubation in 33% methanol on ice. This study bears upon the *in vitro* synthesis of *E. coli* ribosomes (and ultimately self-replication) [17], where the partially sequential, slow modification enzymology is one of the challenges (e.g. see Sup. Figure S8 below)). Nevertheless, a crude cell-free system enables ribosome synthesis and evolution [18].

An *E. coli* strain combining KOs of all 11 ribosomal pseudouridine synthases has been constructed, but the growth and ribosome biogenesis defects were minimal [19]. Recently, we reported the *ΔrluC/ΔrlmE* strain, which lacks psudouridylation of U955, U2504 and U2580 and methylation of U2552 (Figure 1) [5]. This strain displayed the most severe bacterial phenotype yet seen for rRNA modifications: a threefold growth deficit at 37°C and lethality at 20°C. Furthermore, its sucrose gradient profile revealed a severe decrease in the ratio of 70S to 50S. This is consistent with the knowledge that ribosome assembly defects typically display cold sensitivity associated with incorrect rRNA folding [20]. As nucleotide G2553 moved the most in *ΔrlmE* [14], we attributed the surprising potentiation by *ΔrluC* to the closeness of G2553 to one RluC modification site (U2580; see Figure 2G of [5]). We also constructed a second combination KO strain that lacked five modification enzymes, *ΔrluC/ΔrlmKL/ΔrlmN/ΔrlmM/ΔrluE* (abbreviated *ΔCKLNMuE*; note that rlmE is not deleted), resulting in the absence of all modifications in the critical region except D and the partially modified ho5C (Figure 1). Interestingly, it was viable and showed a minimal growth deficit at 37°C [5]. However, at 20°C, it displayed a major growth deficit. Our prior kinetic analyses on these combined KO ribosomes were limited to tripeptide syntheses at 37°C for *ΔrlmE* and *ΔrluC/ΔrlmE*, where WT was 1.3× faster than *ΔrlmE* (consistent with [14]) which was in turn 1.2× faster than *ΔrluC/ΔrlmE* [5]. Our other *in vitro* assays were less comprehensive, either limited to end time points of dipeptide synthesis at 37°C for *ΔrlmE* and *ΔrluC/ΔrlmE* with no differences detected compared to WT, or end time points of fragment reactions on ice in 33% methanol with *ΔCKLNMuE* 50S with a 2× decrease in product yield compared to WT. Furthermore, the kinetic rates were untested at 20°C, the temperature where the most severe growth defects were measured, and the translation rates *in vivo* at any temperature were unknown. Here, we further characterize these strains in order to determine their molecular defects.

**Figure 2.**
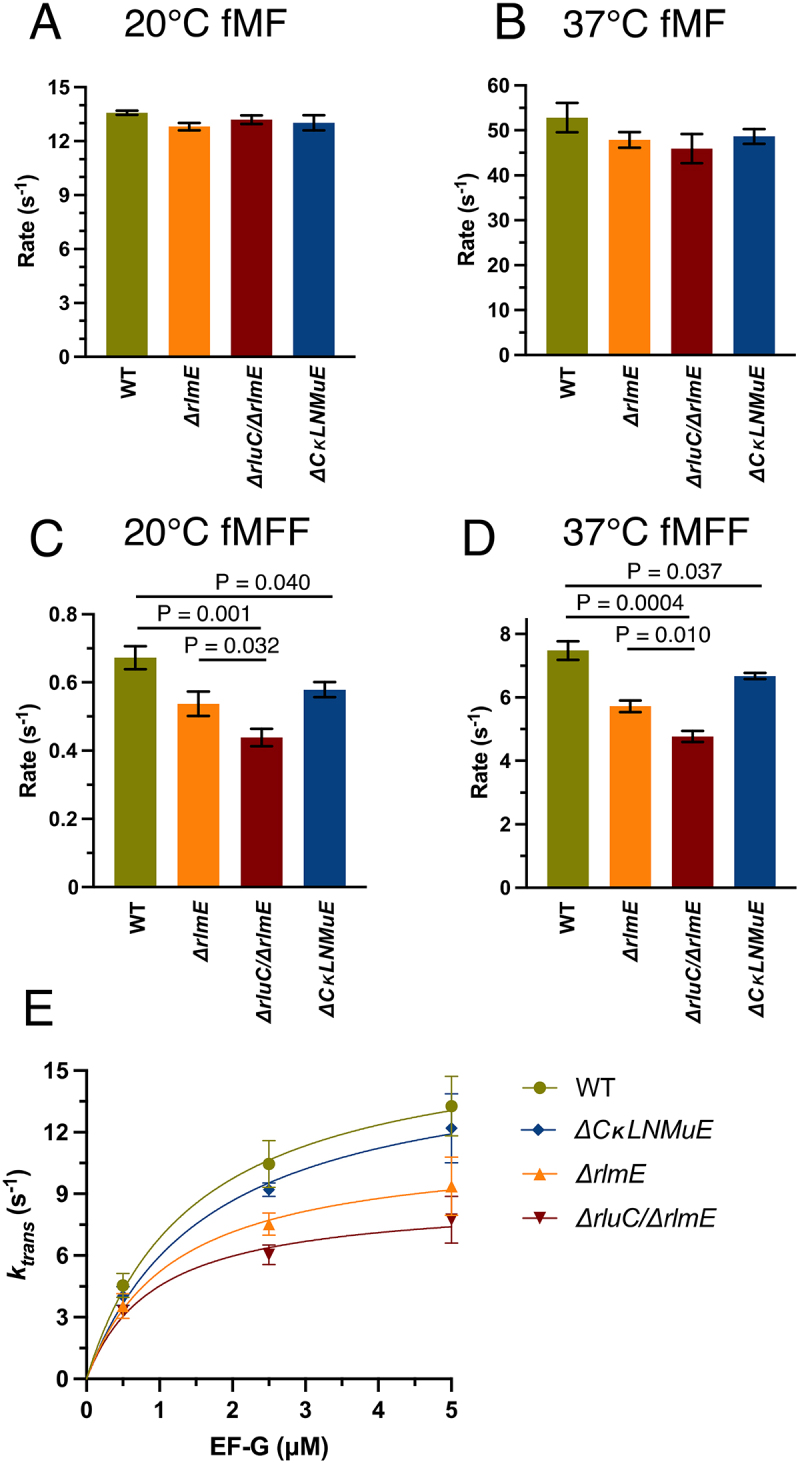
In vitro fast kinetics-based elongation assays of KO ribosomes. fMet-Phe (fMF) dipeptide formation rates at 20°C (A) and 37°C (B), and fMet-Phe-Phe (fMFF) tripeptide formation rates at 20°C (C) and 37°C (D). (E) Calculated translocation rates at 37°C with different EF-G concentrations. Error bars are standard errors, n ≥ 2. p values are not shown for non-significant comparisons. Left three bars in Fig. 2D taken from Liljeruhm et al. (2022).

## Materials and methods

### Materials

Tritium-labelled Met was purchased from PerkinElmer. All other chemicals and reagents were purchased from Sigma-Aldrich or Merck. RNA sequencing reagents and associated methods are cited within [5]. *E. coli* 70S ribosomes, translation factors, and fMet-tRNA^fMet^ were prepared as described [21]. Preparation of the three chromosomal mutant strains *ΔrlmE, ΔrluC/ΔrlmE* and *ΔrluC/ΔrlmKL/ΔrlmN/ΔrlmM/ΔrluE* (abbreviated *ΔCKLNMuE*; note that *rlmE* is not deleted) in MG1655 has been detailed [5]. The gene deletions and absence of pseudorevertants in the 2KO and 5KO strains was concluded from whole-genome sequencing (Eurofins, Germany). MRE600 was used as ‘WT’ for *in vitro* kinetics experiments because it contains lower RNase I activity when compared with MG1655 WT.

### Ribosome sucrose gradients

The sucrose gradient profile was recorded during large-scale ribosome preparation as described [21] with 5 mM Mg^2+^. Briefly, *ΔCKLNMuE* cells were grown to OD_600_ ~0.8 at 37°C in LB medium and collected by centrifugation. The ultracentrifugation-based purification of ribosomes was performed as described [22]. The A_254_ of the sucrose gradient was read with optical unit UV-1 (Pharmacia Biotech) and recorded by BD-112 dual-channel chart recorder (Kipp & Zonen). All *ΔCKLNMuE* 50S subunits used here and in [5] were taken directly from the 50S fraction large peak of the sucrose gradient profile of total ribosomes. WT 50S subunits were purified from the 70S ribosome peak by decreasing Mg^2+^ to 3 mM to allow dissociation of the 50S and 30S, with the 50S fraction being collected by further sucrose gradient ultracentrifugation as for 70S purification.

### Fast kinetics in vitro by quench-flow

Synthetic XR7 mRNA was prepared as described [23] with the sequence: 5′ GGGAAUUCGGGCCCUUGUUAACAAUUAAGGAGGUAUUAA **xxx xxx** UUGCAGAAAAAAAAAAA AAAAAAAAAA3′. The Shine–Dalgarno sequence is underlined and the coding sequences are in bold for fMet-Phe-Phe (AUG UUC UUC) or fMet-stop (AUG UAA). All kinetics experiments were conducted in HEPES-polymix buffer (pH 7.5) containing 5 mM Mg(OAc)_2_, 95 mM KCl, 5 mM NH_4_Cl, 0.5 mM CaCl_2_, 1 mM spermidine, 8 mM putrescine, 1 mM dithioerythritol, and 30 mM HEPES-KOH pH 7.5. Active concentrations of ribosomes (typically 50–75% of the total within the 70S peak on a sucrose gradient for all types of ribosomes here), fMet-tRNA^fMet^, EF-Tu and elongator tRNA^Phe^ were determined through the yield of dipeptide formed when the assayed component was limiting. For dipeptide formation, tripeptide formation and EF-G titration rate assays, 70S initiation complex (IC) and elongation mixture (EM) were formed by preincubating at 37°C for 20 min separately [23]. IC contained 2 μM 70S ribosomes, 5 μM XR7 fMFF mRNA, 2.4 μM f[^3^H]Met-tRNA^fMet^, 2 μM IF1, 2 μM IF2 and 2 μM IF3. For dipeptide formation, EM contained 5 μM EF-Tu, 5 μM tRNA^Phe^, 0.2 mM phenylalanine and 1 μM Phe-tRNA synthetase. For tripeptide formation, EM contained 20 μM EF-Tu, 5 μM EF-Ts, 5 μM EF-G (altered to 1 and 10 μM in EF-G titration assays), 10 μM tRNA^Phe^, 0.4 mM phenylalanine, and 2 μM Phe-tRNA synthetase. Both IC and EM were prepared in HEPES-polymix buffer (pH 7.5) supplemented with 1 mM ATP, 1 mM GTP, 10 mM phosphoenolpyruvate, 0.02 mg/mL pyruvate kinase and 0.005 mg/mL myokinase for energy regeneration. Fast *in vitro* kinetics assays were done at 20°C and 37°C in a temperature-controlled quench-flow apparatus (RQF-3, KinTeck Corp.). Equal volumes of IC and EM were rapidly mixed, and the reactions were quenched with formic acid (17% final) at different time points. The samples were then centrifuged at 20,000 g at 4°C for 30 min and the supernatant removed. Next, 120 μL of 0.5 M KOH was added to the pellets to hydrolyse all the peptides and unreacted f[^3^H]Met from the tRNAs at room temperature for 15 min. Formic acid was added (17% final) to precipitate the deacylated tRNAs. After centrifugation at 20,000 g at 4°C for 30 min, the supernatant was analysed by C18 reversed phase HPLC coupled with a β-RAM model 3 radioactivity detector (IN/US Systems). Separation of f[^3^H]Met-Phe-Phe, f[^3^H]Met-Phe and f[^3^H]Met was achieved by elution with 50% methanol/50% H_2_O/0.1% trifluoroacetic acid for 20 min at 0.45 mL/min. The fraction of [^3^H] peptide out of the total [^3^H] signal at each time point was calculated and the data fitted to a single exponential function with Origin 7.5 (OriginLab Corp.).

For the release assays, release complexes harbouring f[^3^H]Met-tRNA^fMet^ in the P site and the UAA stop codon in the A site were prepared by incubation at 37°C for 20 min of IC mentioned above but with 5 μM XR7 fM-stop mRNA and 2 μM f[^3^H]Met-tRNA^fMet^ instead. This incubated IC was then diluted 10× with HEPES-polymix buffer (pH 7.5) and equilibrated at 37°C for 5 min. The factor mix containing 6 μM release factor 1 (RF1) in HEPES-polymix buffer (pH 7.5) was incubated at 37°C for 5 min. The rates of release were assayed based on *in vitro* quench-flow kinetics as for elongation assays both at 20°C and 37°C. The quenched samples were cooled on ice, and supernatants containing released f[^3^H]Met were separated from pellets containing the f[^3^H]Met-tRNA^fMet^ by centrifugation at 20,000 g for 30 min at 4°C. Pellets were dissolved in 120 μL 0.5 M KOH at 37°C for 15 min, and formic acid was added (17% final) to precipitate the deacylated tRNAs. The radioactive supernatants and dissolved pellets were diluted in ProFlow G+ scintillation liquid for counting (Beckman Coulter LS 6500). The fraction of release was calculated as:

$$\mathrm{Release}\;\mathrm{fraction}={[}^{3}H]\;\mathrm{supernatant}/({[}^{3}H]\;\mathrm{supernatant}+{[}^{3}H]\;\mathrm{pellet})$$

Kinetic rates were estimated by double exponential decay function fitting with Origin 7.5 (OriginLab Corp.). *p* values were calculated using one-tailed *t*-tests.

### In vitro reconstitutions of the 50S subunit

Extractions of 50S total proteins and rRNAs were as described [24] but with ~80 A_260_ units of 50S. The *in vitro* reconstitution was performed as described [24] with minor changes. Briefly, 0.5 A_260_ units of total rRNAs were mixed with 0.6 equivalent units (e.u.) of 50S total proteins (TP50) in a 40 μL total volume containing 20 mM Tris-HCl (pH 7.5), 4 mM Mg(OAc)_2_, 0.4 M NH_4_Cl, 0.2 mM EDTA and 50 mM 2-mercaptoethanol. One A_230_ unit of TP50 = 10 e.u., 1 A_260_ unit of 50S = 1 e.u. TP50. The mixture was then incubated at 44°C for 30 min. Then, the Mg(OAc)_2_ concentration was increased to 20 mM and the solution incubated at 50°C for 90 min. This was then directly used without storage for fragment reactions (see below).

### In vitro peptidyl transferase reactions

Puromycin ‘fragment’ reactions were performed as described [16] with minor changes. Full-length f[^3^H]Met-tRNA^fMet^ (30 pmol) was used as the P-site substrate in a premix (total 60 μL) containing 50 mM Tris-HCl (pH 7.5), 400 mM KCl, 60 mM MgCl_2_, 1 mM puromycin and 15 pmol intact 50S subunit (by A_260_) or 40 μL reconstituted 50S (see above). To this was added 30 μL cold methanol (= 33% final) for incubation on ice for 20 min. The reaction was stopped with 0.4 M final concentration of KOH and incubated at 37°C for 15 min. (This hydrolyses any methanolysis side products; their yield is very minor and much lower than peptide product at all temperatures). Analysis was the same as for *in vitro* elongation assays (see above). The left 2 and right 2 bars in Sup. Figure 4Awere replicated by a different person (results not shown).

### In vivo translation elongation rate assay

The *in vivo* translation rate was measured by β-galactosidase (LacZ) induction as described [25]. Overnight liquid cultures of WT MG1655 (in LB medium) and the KO strains (in LB+Km) were inoculated 1:100 in fresh LB medium (without Km). These were grown to OD_600_ ~0.4 at 37°C and aliquoted into 500 μL/tube. The aliquots were pre-incubated at 20°C or 37°C for at least 10 min. Then, 5 mM IPTG was added to start the induction and, at desired time points, 208 μg of chloramphenicol was added to block further translation. ‘Quenched’ samples were placed on ice and the cells harvested by centrifugation at 20,000 g for 1 min at 4°C. ONPG-based colorimetric assays were performed exactly as described [26] with 0.42 mg/mL chloramphenicol in Z buffer. Miller units were calculated and the LacZ induction time was plotted against square-root of lacZ activity (Schleif plot) to obtain the time for the first LacZ molecule synthesis. The X-axis intercept of the linear part of Schleif plot indicated the time (T_first_) for the ribosome to synthesize the first LacZ protein (1024 amino acids), therefore the elongation rate was calculated as 1024/T_first_.

### Constitutive exogenous protein expression assay

The exogenous reporter expression assays were performed closely to those described [7]. Their assays used plasmid pRFPCERtet with encoded β-lactamase wherein transformed cells were grown with or without anhydrotetracycline. In our assays, MG1655 and KO strains were transformed with pSB1C3 high-copy plasmids that express constitutively amilGFP, mRFP1 or fwYellow [27]. Transformed cells were grown in triplicates for 18 h at 37°C in 1 mL LB media with 33 μg/mL chloramphenicol (without Km). After incubation, the cells were harvested by centrifugation at 20,000 g for 1 min at 4°C and washed twice with PBS. The fluorescence of the cells was measured by an Infinite M200 pro plate reader (Tecan) at 485/535 nm for amilGPF, 531/595 nm for mRFP1 and 503/540 nm for fwYellow. A_600_ was measured at the same time. Fluorescence per cell was calculated by fluorescence/A_600_. All values were normalized to WT. The KO ribosomes were no more sensitive than WT to chloramphenicol in the presence of chloramphenicol acetyltransferase encoded on the pSB1C3 vectors as judged by our protein expression yields.

### RlmE mutagenesis and rescue experiments

Catalytically inert rlmE K38A [10] was recreated by inverse PCR mutagenesis [28] of RlmE in the low-copy pHB plasmid (derived from pSC101 with chloramphenicol resistance) [29]. Oligodeoxyribonucleotide primers (Integrated DNA Technologies) had the sequences: 5'-GCTCTTGATGAAATACAGCAAAGTGAC-3' (forward primer), and 5'-AAACCAGGCACGGGAAC-3' (reverse primer). The plasmids were transformed into WT and *ΔrlmE* MG1655 strains. Bacterial growth was measured with a Spark® 10 M Multimode Microplate Reader (Tecan) and Corning® 96-well flat-bottom plates (Sigma Aldrich).

## Results

### PTC modification enzyme KOs decrease translocation rates at different temperatures

The gaps in knowledge listed in the Introduction concerning the elongation kinetics of our combined KO strains [5] were now filled (Sup. Figure S1 (top); summarized in Sup. Table S1 and Figure 2A–D). Dipeptide syntheses were about 4× slower at 20°C (Figure 2A) than 37°C (Figure 2B) with the different ribosomes giving very similar results (Sup. Table S1). Thus, despite the above-mentioned defect for *ΔCKLNMuE* 50S in the fragment reaction on ice in 33% methanol [5], peptide bond formation rates at 20°C and 37°C in physiological buffers were unaffected in all the KOs and could not explain cold-sensitive bacterial growth. Thus, this defect versus WT in 33% methanol on ice [5] seems irrelevant to the physiological situation (supported also by Sup. Figure S4A discussed below).

Having found significant reductions in tripeptide synthesis rates of the *ΔrluC/ΔrlmE* ribosomes at 37°C [5] that correlated with the major growth deficit of the strain at that temperature, we now tested *ΔCKLNMuE* ribosomes (Figure 2D, blue bar). Indeed, tripeptide synthesis by *ΔCKLNMuE* ribosomes at 37°C was reduced, despite the mild growth defect at that temperature *in vivo*. Given the more severe phenotype of *ΔCKLNMuE* at colder temperature [5], we further tested tripeptide synthesis with *ΔCKLNMuE* ribosomes at 20°C (Figure 2C, blue bar) and again found a defect. The same assays were applied to *ΔrlmE* and *ΔrluC/ΔrlmE* ribosomes (Figure 2C, orange and red bars). Interestingly, although *ΔrluC/ΔrlmE* is lethal at 20°C [5], its 70S ribosomes harvested at 37°C were still able to catalyse tripeptide formation at 20°C at 69% of the WT rate. Compared to *ΔrlmE*, which is viable at 20°C [5], the tripeptide synthesis rate for *ΔrluC/ΔrlmE* was significantly less. These results indicated that slow tripeptide, not dipeptide, synthesis contributes to the cold-sensitivities of all of the KO strains. This conclusion is not unique to fMF and fMFF syntheses as analogous results were obtained for fMI and fMIL syntheses (Sup. Figure S1 (bottom)). This finding that tripeptide synthesis for *ΔrluC/ΔrlmE* was more impaired than for *ΔCKLNMuE* is consistent with the relative growth rates of the strains [5].

To pinpoint whether the binding of translocation factor EF-G or a subsequent translocation step was affected, EF-G was titrated at 37°C and plotted against actual translocation rates calculated as

translocationtime=tripeptidesynthesistime−2×dipeptidesynthesistime

(Figure 2E and Sup. Table S2). For the ribosomes of all strains, curves, rather than straight lines, were obtained that indicated near-saturation at high EF-G concentration (5 μM = 2× standard concentration) and not weaker EF-G binding by the *ΔrlmE* and *ΔrluC/ΔrlmE* ribosomes (based on similar EF-G k_cat_/K_M_ values listed in Sup. Table S2 legend). This suggested that peptidyl-tRNA movement on the ribosome, rather than just weaker EF-G binding, was the affected translocation step (see right graphical abstract image).

Because it cannot be ruled out that the defects are due to possible moonlighting functions of the modification enzymes rather than the absence of rRNA modifications *per se* (see Introduction), we performed two types of experiments here that bear upon this. Firstly, we confirmed the control of Hager et al. [10]; i.e. we reconstructed their catalytically inert RlmE K38A mutant and showed that it could not rescue the growth defect of *ΔrlmE* (Sup Figure 2). Furthermore, we found that expression of the mutant substantially *inhibited* WT and *ΔrlmE* growth (Sup Figure 2). A possible explanation is that the catalytically inert RlmE was trapped on the substrate and thus blocked ribosome assembly. Secondly, we performed five-KO ribosome reconstitutions *in vitro* where the only difference between the tests and controls was the absence of the modifications (not the modification enzymes), yet we still detected catalytic defects (see Figure 4C below). These experiments ([10], Sup. Figure S2 and Figure 4C) support direct roles of the rRNA modifications *per se* in the phenotypes [5] of the KO strains.

### Translation termination at different temperatures is unaffected by PTC modification enzyme KOs

**Figure 3.**
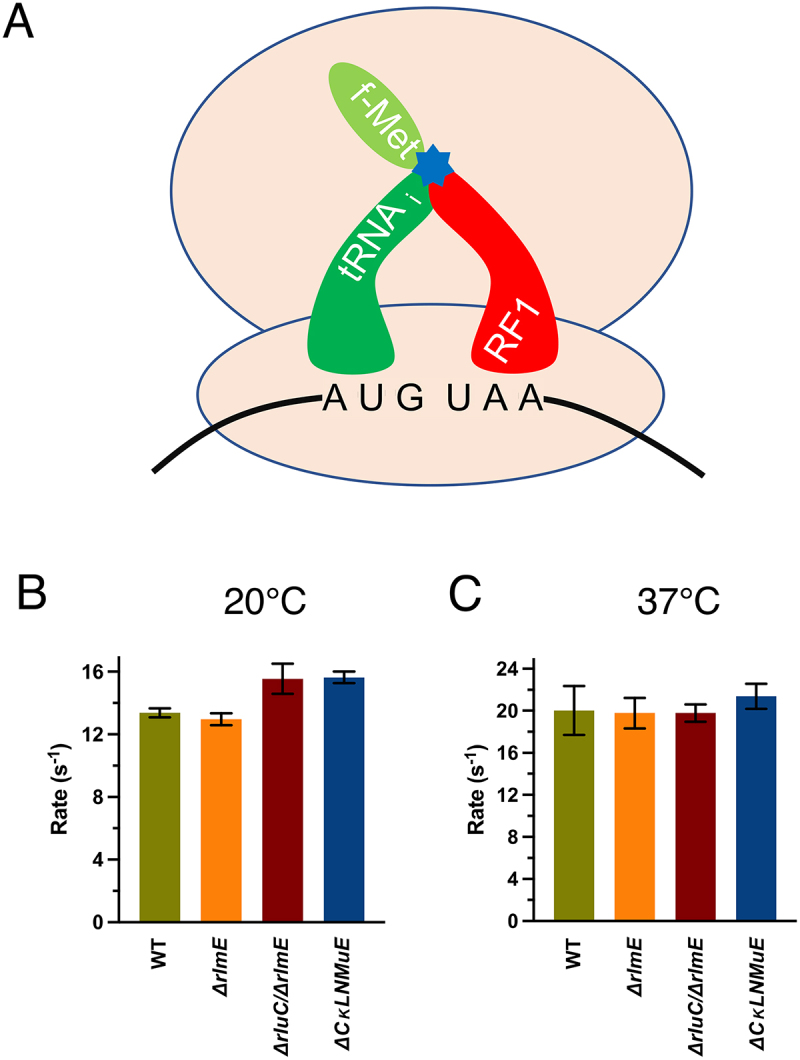
In vitro fast kinetics-based release assays of KO ribosomes. (A) Drawing of release assay depicting RF1-catalyzed hydrolysis (blue star) of the fMet acyl linkage on the initiator tRNA. Rates at 20°C (B) and 37°C (C). Error bars are standard errors, n ≥ 2. Calculated p values > 0.05 based on one-tailed t-test.

In addition to peptide bond formation, the PTC catalyses one other chemical reaction: hydrolysis of the polypeptide from the peptidyl-tRNA in translation termination. We thus wondered whether the modification enzyme deficits would affect a standard release assay (Figure 3A). The release rates were only about 1.3× slower at 20°C (Sup. Figure S3A and Figure 3B) than 37°C (Sup. Figures S3B and S3C) but the different ribosomes gave similar results at a particular temperature. *ΔrluC/ΔrlmE* and *ΔCKLNMuE* displayed slightly faster but insignificantly different release rates compared to WT at 20°C (summarized in Sup. Table S1). This indicated that effects on release did not cause the phenotypes [5] of the KO strains. Taken together, the unaffected dipeptide bond formation and release rates by the modification enzyme KOs measured here imply that the KOs do not substantially affect the PTC-catalysed chemical reactions of the ribosomes around physiological-type conditions.

### KOs of enzymes modifying the critical region impair assembly of the 50S and ribosome

**Figure 4.**
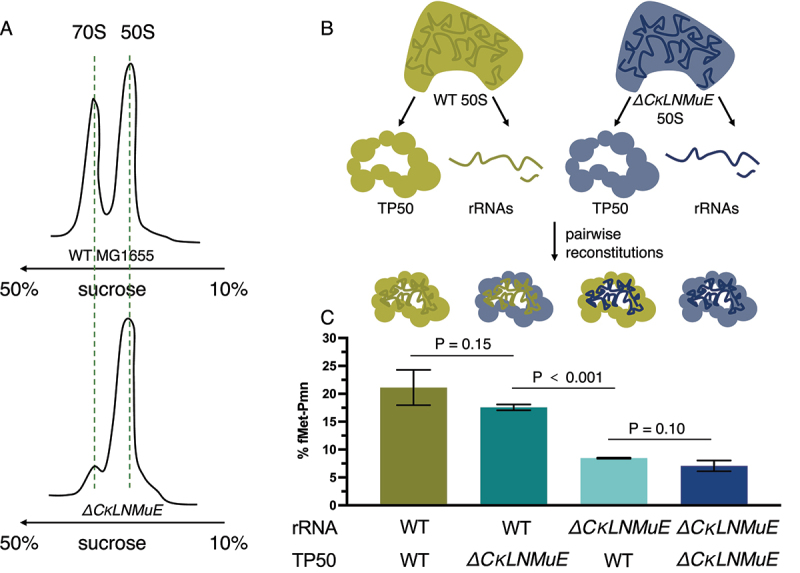
Ribosome sucrose gradient profiles and in vitro reconstitution assays with ΔCKLNMuE. (A) Representative ribosome sucrose gradient profiles at 5 mM Mg^2+^ of ΔCKLNMuE and WT ribosomes. (B) Drawing of extractions of total 50S rRNAs and total 50S proteins and their reconstitutions. (C) Fragment reactions at 37°C of respective reconstitutions drawn directly above. TP50: total 50S proteins. Error bars are standard errors, n = 4.

Our prior sucrose gradient profiling of ribosomes [5] found a major defect in 70S assembly for *ΔrluC/ΔrlmE* but not *ΔCKLNMuE*, despite the latter lacking most of the ‘critical region’ modifications. For *ΔCKLNMuE* ribosomes, growth was rather cold sensitive but 70S assembly was only slightly sensitive to the cold according to the sucrose profiles [5]. However, these conclusions were based on analytical profiles performed using the standard Mg^2+^ concentration of 10 mM, which is unphysiologically high and stabilizing. When we performed large-scale purifications of the 70S ribosomes at 5 mM Mg^2+^, which is closer to the estimated physiological concentration of free Mg^2+^ (1–2 mM) [30], the ratio of 70S to 50S ribosomal particles decreased greatly for *ΔCKLNMuE* compared to the WT (Figure 4A). Although this assay may be ultra-sensitive, in light of the minimal growth defect at 37°C of *ΔCKLNMuE*, the assay result suggests that defective assembly of the 70S ribosome is a major contributor to the cold phenotype of the *ΔCKLNMuE* strain (mindful that its sucrose gradient profiling at 10 mM Mg^2+^ was cold sensitive [5] and that ribosome assembly defects typically display cold sensitivity) [20].

Regarding ribosome assembly without most or all of the critical region modifications, we wondered if our much smaller defect (twofold) in the fragment reaction measured after folding *in vivo* [5] compared with the huge defect after folding *in vitro* (>100× in [16]) was due to *in vivo* versus *in vitro* folding conditions. Armed with sufficient purified 50S subunits from the WT and *ΔCKLNMuE* strains, we prepared total rRNAs and total proteins (TP50) from them for *in vitro* reconstitutions (Figure 4B). Firstly, we titrated the rRNA with TP50 in the standard two-step reconstitution to find that the optimized ratio was 1.0 A_260_ unit of rRNA to 1.2 equivalent units (e.u.) of TP50 (see Methods). The standard fragment reaction (on ice) was used to assay the activity of the reconstituted ribosomes, as well as reactions at 20°C and 37°C to better model physiological conditions. The latter reactions were possible because we found that fragment reactions at 20°C and 37°C were more efficient than those on ice (Sup. Figure S4A; reactions were very slow without methanol (not shown)). At 37°C, the reconstituted *ΔCKLNMuE* 50S showed a threefold decrease in activity compared to the reconstituted WT 50S (Figure 4C, first and last bars) which in turn had half of the activity of purified WT 50S (not shown). To rule out the possibility that the total proteins, rather than the undermodified rRNAs, caused the deficit in activity, we mixed the WT rRNAs with *ΔCKLNMuE* TP50 and *vice versa* (Figure 4C, middle two bars). Indeed, the activities tracked with the rRNAs, not the proteins. Similar results were obtained on ice (Sup Figure S4B). Thus, the major differences between our results and those of [16] cannot be attributed to *in vivo* versus *in vitro* folding (processes which undoubtedly differ and may be influenced by transient binding of modification enzymes) and presumably reflect other differences between the experiments (e.g. different combinations of modifications or their hybridization of non-covalently-linked 23S rRNA pieces). Also, our threefold deficits in the fragment reaction can now be attributed directly to the lack of PTC modifications rather than the lack of modification enzymes, and to impaired folding *in vitro* rather than impaired catalytic chemistry (as the latter is unimpaired at 37°C; see Sup. Figure S4A).

### PTC modification enzyme KOs decrease elongation rates of an endogenous protein in vivo

**Figure 5.**
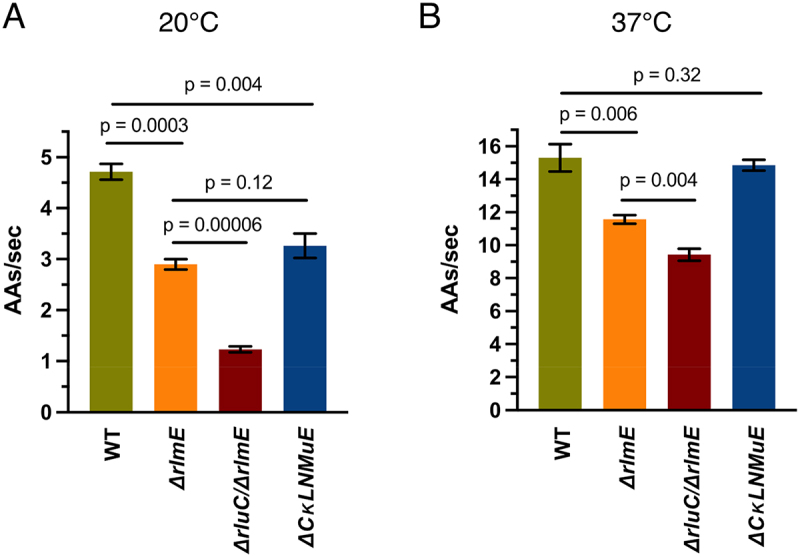
Influence of rRNA modification enzyme KOs on in vivo elongation rates measured by β-galactosidase induction at 20°C (A) and 37°C (B). Error bars are standard errors, n = 3.

Given the slower tripeptide synthesis rates *in vitro* for KO ribosomes ([5,14] and here), we wondered how these correlated with *in vivo* protein synthesis. Therefore, we measured protein synthesis elongation rates *in vivo* using the standard β-galactosidase (*lacZ*) induction assay [25] at 20°C and 37°C. WT and KO strains were grown at 37°C and IPTG was added at the desired temperatures to induce synthesis of β-galactosidase. Our WT elongation rate of 15 amino acids per second at 37°C (Figure 5B) was comparable to published data [31]. At 20°C, WT displayed a 1.5× faster elongation rate than *ΔrlmE* and *ΔCKLNMuE* (Figure 5A and Sup. Figure S5A), correlating with their relative growth rates at that temperature [5]. Notably, *ΔrluC/ΔrlmE* showed a 4.5× slower rate than WT, and 3× slower rate than *ΔrlmE* and *ΔCKLNMuE*, taking ~14 min to synthesize the first β-galactosidase compared with 3.6 min for WT (Figure 5A and Sup Figure S5A). Such slow protein synthesis potentially causes the lethality of the *ΔrluC/ΔrlmE* strain at 20°C (see Discussion). At 37°C, *ΔrlmE* and *ΔrluC/ΔrlmE* showed significantly slower rates than WT, but *ΔCKLNMuE* displayed a similar rate to WT (Figure 5B and Sup Figure S5B). The data correlated well with the *in vitro* tripeptide synthesis rates ([5,14] and Figure 2D) and the minimal deficit in growth rate for *ΔCKLNMuE* at 37°C [5].

### PTC modification enzyme KOs do not affect exogenous protein overexpression

Recently, Pletnev *et al.* [7] reported an *in vivo* protein overexpression assay that found surprisingly significant inhibitions even for most single KOs of *E. coli* rRNA methylation enzymes despite unaffected bacterial growth. Their method was based on overexpression of two fluorescent proteins (FPs) from the GFP family. We therefore tested this method with our KO strains using three related fluorescent proteins, amilGFP, mRFP1 and fwYellow, chosen because their overexpression in *E. coli* occurs without inclusion bodies or high toxicity [27].

Plasmids constitutively expressing the FPs were transformed into *ΔrluC/ΔrlmE* and *ΔCKLNMuE* competent cells and the OD_600_ (Sup. Figure S6A) and fluorescence values were measured after 18 h incubation at 37°C as published [7]. Unexpectedly, FP expression in the KO strains was not reduced compared to WT as judged by fluorescence/OD_600_ (Sup. Figure S6C) or SDS-PAGE which includes measurements of any unfolded FPs (Sup. Figure S6D). Also, the growth rates of *ΔrluC/ΔrlmE* and *ΔCKLNMuE* were suppressed somewhat by overexpression (e.g. for mRFP1, see Sup. Figure S7). Our unexpected finding of reductions in growth rates rather than FP overexpression may reflect unanticipated technical difficulties of this new assay. Also, it makes sense to rely more on the β-galactosidase assay (see above) for *in vivo* measurements because it is well established, has been verified in comparison with other methods (e.g. see Figure S3J of [31]), and it is more physiological than overexpression.

## Discussion

Following up on the unexpected growth phenotypes of both of our combination KOs of enzymes that modify around the PTC [5], the data here provide molecular explanations. For both *ΔrlmE* and *ΔCKLNMuE*, evidence is provided that the growth defects were due to the actual absence of the rRNA modifications, rather than just the absence of the modification enzymes (Figure 4C and Sup Figure 2). As previously noted for *ΔrlmE* [9,12–14] and *ΔrluC/ΔrlmE* [5], a decrease in translocation rate *in vitro* (Figure 2D) and a severe defect in 70S assembly (Figure 4A) is also identified here for *ΔCKLNMuE*. *In vitro* defects in translocation, not dipeptide synthesis or release, are also demonstrated here at the temperature (20°C, Figure 2C) that caused the most severe growth inhibitions for *ΔrluC/ΔrlmE* and *ΔCKLNMuE* [5]. But comparison of the 20°C data with that at 37°C (Figure 2C,D) did not reveal cold sensitivity *in vitro* that could account for the cold sensitivity *in vivo*. However, defects in protein synthesis *in vivo* have now been identified for the KO strains (Figure 5) that correlate with the growth defects at different temperatures [5].

In theory, the % increase in time for a single elongation cycle by the KO ribosomes *in vitro* is expected to cause the same % increase in time for synthesis of the full-length β-galactosidase protein *in vivo* at the same temperature. This is assuming that the measured delay time in synthesis of the 1024-amino-acid β-galactosidase is largely due to elongation, not initiation, which is apparently true for WT (see Figure S3J of [31]). Indeed, there was excellent agreement between the calculated pairs of *in vitro* and *in vivo* elongation cycle times for the three mutants at 37°C (Sup. Table S4). But at 20°C, our *in vivo* times were longer than expected compared with *in vitro* (Sup. Table S4), although the trend compared with WT was the same as at 37°C. Perhaps *in vivo* the translocation defects are potentiated more at 20°C than at 37°C by the major 70S assembly defects associated with the three mutant strains [5] and Figure 4A); this would shift rate limitation in the *in vivo* assay more to initiation (mindful that the *in vitro* values calculated in Sup. Table S4 only include elongation cycle steps). Regardless of the particular steps of protein synthesis affected at 20°C, our *in vivo*-measured large drop from 2.9 amino acids/sec for *ΔrlmE* to 1.2 amino acids/sec for *ΔrluC/ΔrlmE* (Figure 5A) could be responsible for the conditional lethal phenotype of the latter. This conclusion is based on the known correlation between elongation and growth rates at different temperatures and the extremely slow doubling time extrapolated from around 1.2 amino acids/sec on Figure 3 in [32].

It is puzzling that mature 70S ribosomes from our strains with combination KOs of enzymes that modify around the PTC both exhibited a defect, not in the two PTC functions of peptide bond formation and release, but rather in translocation. Similar kinetics results were published by others for *ΔrlmE* [14]. The ratcheting movements between the subunits that occur during translocation are distal from the PTC. However, a structural study of mature 50S particles from the *ΔrlmE* strain revealed that the largest rRNA displacements were of U2554 and G2553 [14], the latter being the A-site nucleotide which base pairs with the CCA end of aminoacyl-tRNA (immediately adjacent to Um2552 as shown in Figure 1) during translation [33]. After peptide bond formation occurs, the translocation steps include breaking this base pair. But dipeptide formation requires the formation of this base pair, and it is unknown if the U2554 and G2553 displacements occur in mature 70S from *ΔrlmE*. Another consideration is that, in contrast to translocation, both dipeptide formation and release are single-round nucleophilic attacks catalysed by the PTC without as large a conformational change of the ribosome as occurs in translocation ratcheting [34,35], so translocation may be more sensitive to structural changes.

Also hard to explain is defective 70S formation in all three KO strains, given that the PTC region is distal from the 50S subunit interface (see left graphical abstract image). However, an assembly deficit seems reasonable based on the ribosomal literature (e.g. [14,20,36,37]). Ribosome assembly defects typically display cold sensitivity [20], *ΔrlmE* cells that lack a modification near the PTC accumulate pre50S particles with rRNA structural differences and ribosomal protein absences that cluster near the PTC [14], and PTC formation is the final step in 50S maturation necessary for binding to the 30S [36,38]. After PTC maturation, no further conformational change in the 50S is required for 70S formation because the 50S has the same 3D structure whether or not it is bound to the 30S [39]. Although our 5KO 50S preparation that failed to form 70S functions quite well in fragment reactions here (with or without prior reconstitution), this can be rationalized by the use of unphysiologically high Mg^2+^ concentrations which are known to facilitate PTC maturation. For *E. coli* 50S reconstitutions *in vitro*, a heat activation step at unphysiologically high Mg^2+^ concentration is needed to fold the disordered rRNA within the peri-PTC region of helices H90-H93 (Figure 1) [40].

In summary, different KO combinations around the PTC, though differing in phenotypic severity, exhibited remarkably similar molecular defects in translation. Evidence indicates that the enigmatic PTC region modifications act in synergy to correctly fold domain V of the 50S to allow efficient 70S assembly and translocation. However, the functions of these modifications cannot be general, as only Um2552 and Ψ2457 are nearly universally conserved [41].

## Supplementary Material

20240130 Sup Figs and Tabs.docx

20240604 revised Sup.docx

## Data Availability

The data supporting the study are presented in the figures and tables in the main text and the supplementary material and are available in more detail upon request from LB and AF.
