# Supplementary material for: Translational impacts of enzymes that modify ribosomal RNA around the peptidyl transferase centre
Source: RNA Biol. 2024 Jul 1;21(1):31–41. doi: 10.1080/15476286.2024.2368305 (PMC11221467; doi:10.1080/15476286.2024.2368305)
Supplement: 20240604 revised Sup.docx [file KRNB_A_2368305_SM7422.docx]

SUPPORTING INFORMATION

**Translational effects of enzymes that modify**

**around the peptidyl transferase center**

**Letian Bao^a^, Josefine Liljeruhm^a^, Rubén Crespo Blanco^a^, Gerrit Brandis^a^,**

**Jaanus Remme^b^ and Anthony C. Forster^a,1^**

^a^Department of Cell and Molecular Biology, Uppsala University, Husargatan 3, Box 596, Uppsala 75124, Sweden;

^b^Department of Molecular Biology, University of Tartu, 51010 Tartu, Estonia.

^1^To whom correspondence may be addressed. E-mail: [a.forster@icm.uu.se](mailto:a.forster@icm.uu.se)

Contents

**Table S1.** Summary of translation and release rates of KO ribosomes at 20 and 37℃ for Figs. 2A-D & 3.

**Table S2.** Summary of calculated translocation rates of KO ribosomes at 37℃ for Fig. 2E.

**Table S3.** Summary of *in vivo* β-galactosidase synthesis rates of KO ribosomes at 20 and 37℃ for Fig. 5A and B.

**Table S4.** Comparison of fold changes (normalized to WT) of times for a single elongation cycle *in vitro* (calculated as τ*_fMFF_* – τ*_fMF_* with 2.5 μM EF-G from Sup. Table 1) and *in vivo* (calculated as 1/(amino acids/sec) from Fig. 5).

**Figure S1.** *In vitro* fast kinetics-based elongation assays of KO ribosomes.

**Figure S2.** Rescue experiments by expressing functional or catalytically-inert RlmE enzymes in WT or *ΔrlmE* strains.

**Figure S3.** *In vitro* fast kinetics-based release assays of KO ribosomes.

**Figure S4.** Comparison of fragment reactions at different temperatures.

**Figure S5.** Time courses of β-galactosidase induction *in vivo* (Schleif *et al.*, 1973) at 20℃ (A) and 37℃ (B).

**Figure S6.** Influence of rRNA modification enzyme KOs on overexpression of fluorescent proteins and on growth.

**Figure S7.** Influence of constitutive overexpression of mRFP1 on growth of *ΔCKLNMuE* and *ΔrlmE/Δrlu*C.

**Figure S8.** Reconstitution *in vitro* of 4 simultaneous efficient modifications of the 23S rRNA critical region using subcloned genes for the rRNA and modification enzymes.

**Graphical abstract**

**Sup. Table 1.** Summary of translation and release rates of KO ribosomes at 20 and 37℃ for Figs. 2A-D & 3.

**Sup. Table 2.** Summary of calculated translocation rates of KO ribosomes at 37℃ for Fig. 2E. Values at 20℃ are included for comparison. Calculated k_cat_/K_m_ values at 37℃: WT, 11.5; *ΔrlmE*, 9.6; *ΔrluC/ΔrlmE*, 9.3 and *ΔCKLNMuE*, 9.5.

**Sup. Table 3.** Summary of *in vivo* β-galactosidase synthesis rates of KO ribosomes at 20 and 37℃ for Fig. 5A and B.

**Sup. Table 4.** Comparison of fold changes (normalized to WT) of times for a single elongation cycle *in vitro* (calculated as τ*_fMFF_* – τ*_fMF_* with 2.5 μM EF-G from Sup. Table 1) and *in vivo* (calculated as 1/(amino acids/sec) from Fig. 5).

**Sup. Figure 1 (top).** *In vitro* fast kinetics-based elongation assays of KO ribosomes. fMet-Phe dipeptide formation time courses at 20℃ (A) and 37℃ (B), and fMet-Phe-Phe tripeptide formation time courses at 20℃ (C) and 37℃ (D). Error bars are standard errors.

**Sup. Figure 1 (bottom).** *In vitro* fast kinetics-based elongation assays of KO ribosomes. fMet-Ile dipeptide formation time courses (E) and rates (F), and fMet-Ile-Leu tripeptide formation time courses (G) and rates (H) at 37℃. Error bars are standard errors.

**Sup. Figure 2.** Rescue experiments by expressing functional or catalytically-inert RlmE enzymes in WT or *ΔrlmE* strains. The generation times were plotted. pHB is the low-copy plasmid backbone. The WT pHB grew more slowly than expected for a WT *E. coli* strain, perhaps due to the HB plasmid. Error bars are standard errors, n ≥ 7. One-tailed P = 0.0025 for WT pHB vs. *ΔrlmE* pHB; 0.039 for *ΔrlmE* pHB vs. *ΔrlmE* pHB-rlmE; 0.44 for WT pHB vs. *ΔrlmE* pHB-rlmE.

**Sup. Figure 3.** *In vitro* fast kinetics-based release assays of KO ribosomes. Time courses at 20℃ (A) and 37℃ (B). Error bars are standard errors.

**Sup. Figure 4.** Comparison of fragment reactions at different temperatures. (A) Intact WT and *ΔCKLNMuE* 50S-catalyzed fragment reactions under standard conditions (on ice) or at higher temperatures. (B) Pairwise reconstituted 50S-catalyzed fragment reactions on ice for 20 min. Controls without 50S gave negligible signal. Error bars are standard errors, n ≥ 2.

**Sup. Figure 5.** Time courses of β-galactosidase induction *in vivo* (Schleif *et al.*, 1973) at 20℃ (A) and 37℃ (B). E = enzyme activity. The X-axis intercepts of the linear parts of the Schleif plots indicated the time (T_first_) for the ribosome to synthesize the first LacZ protein (1024 aa), so the elongation rates (see Fig. 5) were calculated as 1024/T_first_.

**Sup. Figure 6.** Influence of rRNA modification enzyme KOs on overexpression of fluorescent proteins and on growth. (A) Growth of the respective strains at 18h. (B) Constitutive overexpression at 37℃ of functional green (amilGFP), red (mRFP1) and yellow (fwYellow) fluorescent proteins encoded on high-copy plasmids. (C) Normalized fluorescence per cell. Error bars are standard errors, n = 6. (D) Gel analysis of influence of rRNA modification enzyme KOs on the total yield of overexpressed amilGFP, mRFP1 and fwYellow. Representative SDS-PAGE gels after 18h incubation at 37℃ show both functional and non-functional constitutively-expressed Coomassie-stained proteins. Cells from the same culture volumes were loaded in each lane. Arrows indicate induction bands.


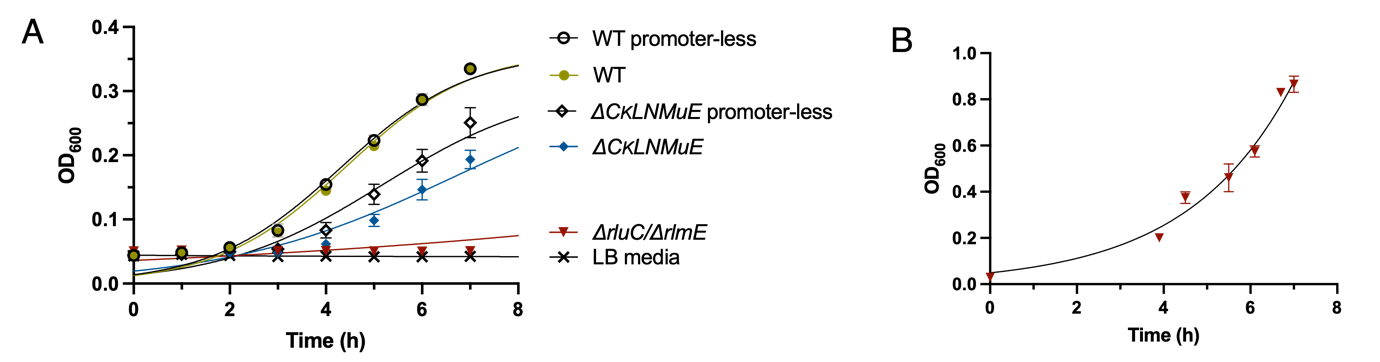


**Sup. Figure 7.** Influence of constitutive overexpression of mRFP1 on growth of *ΔCKLNMuE* and *ΔrlmE/Δrlu*C. (A) Growth of two combined KO strains when harboring mRFP1 encoded on a high-copy plasmid. In two controls, the plasmid lacked the promoter for mRFP1 (promoter-less). (B) Growth of *ΔrlmE/Δrlu*C without plasmid at 37℃. Different OD ranges were due to measurement with plate reader or spectrophotometer. Error bars are standard errors, n ≥ 3.

**
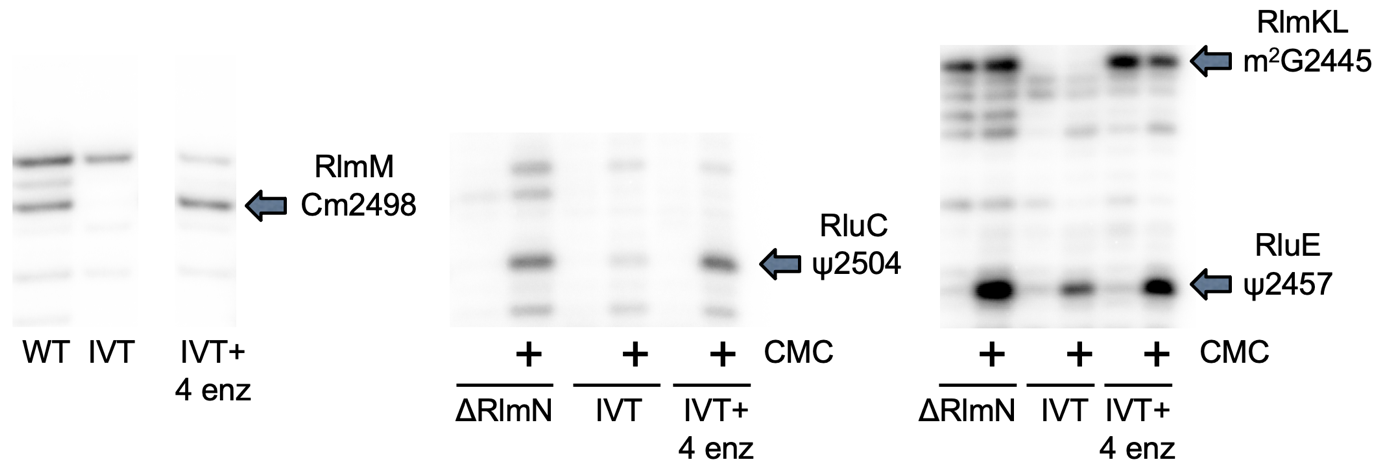
**

**Sup. Figure 8.** Reconstitution *in vitro* of 4 simultaneous efficient modifications of the 23S rRNA critical region using subcloned genes for the rRNA and modification enzymes. The 23S rRNA gene was transcribed by T7 RNA polymerase for 2h in the presence of 0.5 μM each of overexpressed RlmM, RluC, RlmKL and RluE enzymes (4 enz) and 0.2 mM *S*-adenosyl methionine, then split into aliquots for analysis. Some aliquots were treated with CMC reagent to chemically modify pseudouridines to enable their detection. Gel analysis showed, at all 4 possible modification positions within the critical region, pausing by reverse transcriptase (arrows) was much greater than for the negative-control *in vitro* transcript (IVT) and was comparable to the positive controls (WT or ∆RlmN 23S rRNAs).

**Graphical abstract**
